# High-grade spindle cell sarcoma of the heart: a case report and review of literature

**DOI:** 10.1186/s13019-015-0245-6

**Published:** 2015-03-29

**Authors:** Alex Muturi, Vihar Kotecha, Josiah Ruturi, Morris Muhinga, Wairimu Waweru

**Affiliations:** 1 Department of Surgery, University of Nairobi, Nairobi, Kenya; 2 Department of Cardiothoracic Surgery, Kenyatta National Hospital, Nairobi, Kenya; 3 Department of Pathology, University of Nairobi, Nairobi, Kenya

**Keywords:** Right ventricle, Spindle cell sarcoma, Myxoma

## Abstract

Primary cardiac spindle cell sarcomas are extremely rare and have poor prognosis. Complete surgical resection represents the only treatment option proven to work. We report a case of a 28-year-old man who was referred to our cardiothoracic unit with a right ventricular mass extending to the main pulmonary artery with a provisional diagnosis of a thrombus or a right ventricular myxoma. He sought medical attention after experiencing shortness of breath, cough, chest pain, abdominal pain and bilateral leg swelling for about 6 weeks. Two dimensional transthoracic echocardiogram showed a mass arising from the right ventricle and extending into the main pulmonary artery; findings that were confirmed with contrast chest CT scan.

He underwent extensive resection of the mass and had uneventful postoperative period with immediate symptomatic relieve. He is on adjuvant chemotherapy using vincristine, adriamycin and cyclophosphamide (VAC). Right ventricular sarcoma is a very rare cause of right sided heart failure, a very aggressive tumour whose only chance of successful treatment is complete surgical excision.

## Background

Primary cardiac tumours are rare with a reported incidence of 0.017-0.019 [1]. Most cardiac tumours are metastases with majority having a pulmonary source [2]. Seventy-five percent of primary cardiac tumours are benign with close to half being myxomas [1,3]. Twenty-five percent are malignant with 95% of these being reported as sarcomas [1]. The most common sarcoma is angiosarcoma (34%) and undifferentiated sarcoma second at 24%. Others include rhabdomyosarcoma, osteosarcoma, synovial sarcoma and leiomyosarcoma [1]. The least reported cardiac tumours are spindle cell sarcomas [1,4-6].

These tumours are rare and produce non-specific symptoms. They are thus often difficult to diagnose preoperatively and they can be missed. Use of echocardiogram (transesophageal echocardiogram is more sensitive than transthoracic Echo), CT scan and cardiac MRI makes preoperative diagnosis possible. In most cases, the provisional diagnosis is a benign myxoma and the suspicion of sarcomas is only made intraoperatively due to the tendency of the tumour to be invasive. Due to rarity of these tumours, there is no consensus on effective adjuvant therapy.

## Case presentation

A 28 year old mechanic from rural Kenya, was referred to our unit with a provisional diagnosis of right ventricular tumour, favouring either a thrombus or myxoma. He sought medical attention after experiencing shortness of breath, cough, chest pain, abdominal pain and bilateral leg swelling for 6 weeks. He also reported unexplained weight loss (8 kg), fever and night sweats. He had been admitted two weeks earlier at a peripheral hospital and treated for pneumonia, but the symptoms persisted. Tests for tuberculosis were negative. There is no history of asthma in the family. He doesn’t smoke or take alcohol. He is single and lives with his parents.

Physical examination revealed marked bilateral pedal oedema, distended neck veins, and tender hepatomegaly. The laboratory tests showed markedly elevated liver enzymes; AST 664 (5–42) IU/L and ALT 224 5–42) IU/L with other blood tests being within normal limits. Based on these features of right-sided heart failure and suspicion of a thrombus in the right ventricle, he was started on heparin, frusemide and spirinolactone with modest symptomatic improvement.

**Figure 1:**
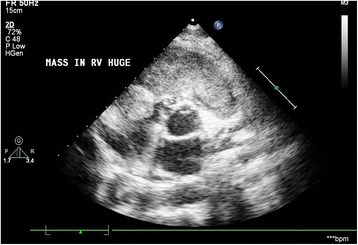
**Preoperative echocardiogram showing right ventricular mass.**

A chest x-ray showed cardiomegaly and minimal right sided pleural effusion. The ECG revealed sinus tachycardia and right ventricular hypertrophy. Two Dimensional transthoracic echocardiogram showed a mass arising from the right ventricle and extending into the main pulmonary artery, small pericardial effusion and a right sided pleural effusion. Contrast Chest CT scan confirmed the Echo findings (Figure 1).

Intraoperatively we found, normal appearing pericardium, and minimal pericardial effusion. Right ventriculotomy was performed after going on cardiopulmonary bypass, we found a whitish yellow lobulated, friable mass that appeared to arise from the septal leaflet of the tricuspid valve, with a wide base and filling the entire ventricular chamber with diffuse infiltration of the ventricular walls and extending into and nearly occluding the main pulmonary artery trunk, without adhering to it.

With pulmonary artery cross clamp on, the mass was gently delivered from the pulmonary artery and dissected off the RV and the tricuspid valve. The tricuspid valve was then repaired. Due to the diffuse infiltrating nature of the tumour, resection with margins was not feasible. The patient was nursed in ICU and discharged to the general ward on the third postoperative day. Repeat echocardiogram in the immediate postoperative period showed moderate tricuspid regurgitation, mild pulmonary hypertension and a mild right-sided pleural effusion.

He was put on diuretics; frusemide and spirinolactone and he is currently asymptomatic. Definitive histopathology of the mass revealed a high-grade spindle cell sarcoma. Immunohistochemical tests showed the tumour only had patchy positivity for AE1/AE3 and negative for the following markers: SMA, Desmin, CD34, S100, EMA, and CD99. Staining for CD117 was not performed.

He is undergoing adjuvant chemotherapy using VAC regimen. He received 2 mg of Vincristine as the total dose per course, Adriamycin at 70 mg/m^2^, and Cyclophosphamide at 600 mg/m^2^, administered as intravenous bolus infusion. He received six courses of adjuvant chemotherapy at 21 days interval.

## Discussion

This patient was first admitted with a diagnosis of a thrombus in the right ventricle and started on anticoagulation and ant failure regime. After ten days of treatment without much improvement the cardiothoracic team was consulted and possibility of a heart tumour was encouraged, a repeat two-dimensional transthoracic echocardiogram still showed a mass in the right ventricle.

Our facility doesn’t have capability to perform transesophageal echocardiogram which would better define the intracardiac mass. The patient went to surgery with a provisional diagnosis of right ventricular myxoma or a thrombus, these being the commoner intracardiac tumours. It was not possible to achieve tumour free margins due to the extensive infiltration of the tumour into the right ventricular walls. The post operative echocardiogram showed marked improvement in cardiac output and right ventricular function.

The patient was started on adjuvant chemotherapy, using the VAC regimen, which in our set up is the affordable treatment option for sarcomas. This patient is being followed up at our cardiothoiracic outpatient clinic every three months, oncology unit and cardiology clinic. In view of the intraoperative findings and the chemotherapy regimen he is on that includes Adriamycin, an echocardiogram will be performed at the end of each treatment cycle to reassess the functional status of the heart. The most recent echocardiogram done three months after surgery, showed mild tricuspid regurgitation, he remains symptom free and he is off ant failure drugs.

The earliest report of tumour in the heart dates back to mid 16th century, by Colombus [7]. The commonest cardiac sarcomas are angiosarcomas, followed by malignant fibrous histiocytomas (MFH). The angiosarcomas have predilection for the right side of the heart with the MFHs favouring the left [3,4] Cardiac spindle cell sarcomas are tumours of mesenchymal origin, more commonly affecting the large blood vessels. They are extremely rare in the heart, with only four cases reported so far [4-6]. Echocardiography by transthoracic or preferably transesophageal route is easy to perform, rapid and inexpensive tool to identify intracardiac masses. Transesophageal echocardiography (TEE) provides better resolution, as it allows for use of higher-frequency transducers. However, TEE provides limited soft-tissue characterization and visualization of the mediastinum [2]. CT and MRI scan of chest and abdomen complement echocardiography [8].

Unlike echocardiography, CT and MRI have the advantage of showing the extra cardiac extent of tumour and presence of metastases, myocardial infiltration, compression of cardiac chambers along with pericardial and great vessel involvement [8]. Cardiac MRI (CMRI) is useful to assess tumour volume, tumour burden, mediastinal invasion and response to therapy. The limitations of CMRI include cost and dependence on regular electrocardiographic rhythms and cardiac gating [8]. Chest x-ray though not diagnostic may show cardiomegaly, infiltrates suggestive of pulmonary oedema due to congestive cardiac failure, pleural effusion, lung nodules, cardiac mass or left hemi diaphragm paralysis [2]. Electrocardiogram is usually non-diagnostic being normal or may show non-specific changes such as conduction block, right ventricular hypertrophy, atrial fibrillation, paroxysmal atrial tachycardia etc. [2]. Where expertise exists and condition of the patient permits, transvenous end myocardial biopsy is helpful to yield a histological confirmation before operation [9].

These tumours are highly aggressive, rapidly infiltrating all the layers of the heart and metastasize rapidly. At the time of presentation up to 80% have evidence of metastasis [10]. The prognosis of cardiac primary spindle cell sarcomas is poor because these tumours are highly aggressive with reported mean survival of approximately 3 months to 1 year. Survival of up to 11 years has been reported [6]. Cardiac tumours can cause significant morbidity and mortality because of the delicate structures involved [4].

The effects of a cardiac tumour depend on its anatomical location in the heart, size, invasiveness, friability, and the rate of growth with the most important factor affecting the prognosis of these tumours being the anatomical location in the heart (intracavitary versus intra/extramyocardial growth) [4]. Surgery with negative resection margins is the only proven successful treatment so far but complete tumour resection is only possible in less than 50% of patients [4]. It has been shown that patients who undergo surgery with negative resection margins have two times the life expectancy of those who complete surgical resection is not feasible [11], this shows that early diagnosis and timely treatment have an impact on treatment success and prognosis. Additionally surgery also has a role in palliative resections for relief of symptoms, biopsy to confirm diagnosis or repeated resections. Though the initial results of surgery are encouraging their long-term survival remains poor mostly due to local or systemic recurrence. Never the less surgery has been shown to prolong the survival and improve the quality of life [11].

When spindle cell sarcoma arises in the heart, the differential diagnosis includes angiosarcoma or synovial sarcoma. Spindle cell sarcomas usually show positive immunoreactivity for vimentin, osteopontin, and MDM2 [12,13]. Variable positivity may be observed for alpha smooth muscle actin, CD117, CD68, p53, and bcl-2. Occasionally, the tumour has some positive staining with antibodies against desmin. CD31, CD34, and Factor VIII are typically negative. The role of chemotherapy or radiotherapy in the treatment of primary cardiac sarcoma has not proven to be beneficial [14], though there are some reports of adriamycin [14] and doxorubicin [15] showing some improvement in survival. Poor tolerance of radiation by the heart has limited the use of radiotherapy. Though it’s role has not been proven, radiotherapy has been used for treatment of positive margins after resections, palliation of aggressive localized disease and local recurrences [4].

## Conclusion

Cardiac sarcomas remain a challenge to treat partly due to their rarity and also because of the aggressive nature of these tumours. Its diagnosis can be challenging due to the nonspecific nature of presentation. In our setup two-dimensional echocardiography is available and this tool aids in quick evaluation of the heart enabling the genesis of a prompt provisional diagnosis. Surgery remains the mainstay of treatment. Keeping in mind that adjuvant therapy has poor response the cardiac surgeon needs to strike a balance between obtaining adequate tumour free margin and leaving behind a functioning heart.

## Consent

Informed consent was obtained from the patient for publication of this case report.

## Abbreviations

AE1/AE3: Antigen E1 and E3 (multi-cytokeratin)
ALT: Alanine transaminase
AST: Aspartate transaminase
Bcl-2: B-Cell Lymphoma 2 gene
CD: Cluster of differentiation
CT Scan: Computed tomography scan
ECG: Electro cardiogram
EMA: Epithelial membrane antigen
IU/L: International units per litre
MDM: Murine double minute
MFH: Malignant fibrous histiocytoma
MRI: Magnetic imaging resonance
RV: Right ventricle
SMA: Smooth muscle actin
TEE: Trans-esophageal echocardiogram
